# The impact of community-based interventions for the older population: a quasi-experimental study of a hip-fracture prevention program in Norway

**DOI:** 10.1186/s12877-018-1004-z

**Published:** 2018-12-13

**Authors:** Henning Øien, Niklas Jakobsson, Carl Bonander

**Affiliations:** 1 Centre for Public Safety, Karlstad University, Karlstad, Sweden; 2 Health Metrics Unit, Sahlgrenska Academy, University of Gothenburg, Gothenburg, Sweden; 3 Karlstad Business School, Karlstad University, Karlstad, Sweden; 4 Norwegian Social Research, Oslo Metropolitan University, Oslo, Norway

**Keywords:** Hip fracture, Community interventions, Natural experiments, Difference-in-differences, Matching

## Abstract

**Background:**

Hip fractures among older adults are a major public health problem in many countries. Hip fractures are associated with expensive health care treatments, and serious adverse effects on patients’ health and quality-of-life. In this paper, we estimate the effect of a community-based hip fracture prevention program that was initiated in 16 Norwegian municipalities in 2007. Specifically, the participating municipalities implemented one or more of the following interventions: exercise programs for older adults, information and education campaigns to communicate how to effectively reduce falls to care workers and older adults, and preventive home safety assessment and modification help services.

**Methods:**

We used a difference-in-difference design, and identified control municipalities by matching on pre-intervention trends in the outcome. The outcome measure was the incidence of hip-fractures among older adults (≥65 years).

**Results:**

We found no statistically significant effects of the implemented program on the incidence of hip fractures, on average, in older subgroups (≥80 years) or in municipality-specific analyses.

**Conclusions:**

It is unclear whether the interventions managed to achieve a change in hip fracture rates at the population level.

## Background

Falls are one of the leading causes of unintentional injury hospitalizations and deaths from injury worldwide [15]. Falls among older adults are common [10, 33, 39], and the risk of falling increases even further with higher age [45]. Hip fractures are one of the most serious fall-related injuries in terms of excess mortality and morbidity [19, 20]. The risk of experiencing a hip fracture after a fall is higher among older adults due to age-associated risk factors such as osteoporosis [8, 25]. Beyond individual-level health losses, hip fractures are also associated with large economic losses to society due to excess hospital costs [25]. Meanwhile, the burden of hip fractures is also expected to increase in the future due to demographic shifts towards an aging population [7, 37].

The Scandinavian countries are among the countries with the highest incidence of hip fractures [21], and in Norway the incidence was reported to be particularly high in the 1990s [26]. The high monetary and quality of life costs associated with hip fractures put pressure on public health officials to introduce public health policies that effectively reduce hip fractures. Evidence from meta-analyses of randomized controlled trials indicates that exercise programs and home hazard modification can decrease falls and hip fracture rates [11, 22]. There is, however, lack of evidence regarding the population-level impact of community-based interventions [6, 29, 31]. Hence, successful results found in experimental settings does not seem to be transferable to the community level. McClure et al. [30] argue that the reason behind this is that interventions studied in experimental settings often focus on isolated, component causes of injury without considering complex social causes and governance.

The systematic approach to prevention entails problem surveillance, risk factor identification, development of interventions in controlled settings, and finally implementing and scaling up interventions that are identified as successful and cost-effective [30]. While the first three steps are often achieved with high levels of scientific rigor, the quality of evidence of the effectiveness of scaling interventions up to the community level is lacking due to limitations of conducting randomized trials at the community level [41]. We have only found one paper conducting a randomized controlled community trial of a fall prevention program [12]. They studied the effects on fall injuries of an enhanced multifaceted support system for communities in Wisconsin, US. The supported system included, among other interventions, technical assistant, capacity building and support in community. There were clear reductions in fall injuries in the communities that adapted the support system compared to the control communities. While randomization is regarded as the most internally valid method for reducing confounding bias, community-based trials are often restricted to a small sample of communities, meaning that imbalance on unobservable confounders may remain. For example, Guse et al. [12] included only 20 communities and two treatment arms. In the analysis they control for confounders, which do not change the main results. However, this does not guarantee that unobservable factors might confound the analysis.

When studying the effects of interventions that are initiated by societal actors, randomization is often considered unfeasible or impossible. Instead, we must often turn to quasi-experimental alternatives to deal with confounding bias. A common approach in previous studies of community-based falls and hip fracture programs has been to use a controlled before-after design in which the changes in the intervention community is compared to that of similar or proximate communities. A systematic review of population-based hip fracture prevention programs identified only five controlled before-after studies that met their methodological criteria [31].^1^

A popular solution to the problem of causal inference in similar settings is to use a difference-in-differences (DD) design, which identifies the causal effect of the intervention under the common trends assumption [3]. None of the included papers used this approach, nor did they attempt to validate this assumption by checking for historical trends. More recent approaches to DD methods focus on propensity score matching on pre-intervention covariates or use re-weighting to generate so-called synthetic controls based on a larger pool of untreated units (or potential controls) [1, 2]). However, while the average bias tends to decrease by propensity score matching [42], it requires the selection and measurement of important control variables, some of which may be unobservable, unknown or unavailable. In our experience, synthetic control methods also tend to work poorly with incidence rate data from small communities (more generally, any noisy time series), as we run the risk of matching on random error rather than the signal of the trend [1]. This calls for an approach that (1) identifies the best controls empirically, (2) does not require the use of covariates and (3) uses cross-validation methods to avoid matching on random error [38]. In this paper, we use such an approach to identify the effects of a set of hip fracture prevention programs that were implemented in 15 Norwegian municipalities in 2007, selecting our control communities by matching on pre-intervention trends. The programs are aimed at preventing falls and fall-injuries among the older adults (≥65 years) by implementing a wide range of countermeasures aimed at changing attitudes, behavior, and the physical and organizational environment. Hence, they serve as interesting case studies of community-based implementations of hip fracture prevention measures.

## Methods

### Setting

In Norway, the municipalities have the overall responsibility of residents’ health [14]. The municipalities are obligated by law to promote health, and prevent injuries, accidents and social problems among its citizens. They are also responsible for funding and providing necessary primary care to their residents.^2^ Necessity is defined by the health needs of residents. Municipalities are restricted to allocate services according to health needs and independently of socioeconomic status [23]. Among the primary care services are all social and community health services provided to persons with long-term care (LTC) needs. The LTC services the municipalities finance and provide can be broadly divided into nursing and home-based care services. The LTC sector can be said to be semi-centralized [13]. In the sense that the central government determines the legal bounds of municipalities’ health and care responsibility, while the municipalities have extensive discretion in determining the composition of preventive, long-term, and curative care that best meet the needs of their residents.

LTC expenditures is the largest component of municipal spending [14]. There is a fear that this funding responsibility will become more demanding in the future because of an aging population. This has led many municipalities to focus more on measures that can prevent and postpone care needs. One such measure is interventions to prevent hip fractures. Hip fracture prevention efforts have received a lot of attention in Norway. This is because Norway has one of the highest hip fracture incidence rates in the world [21, 28], and experiencing a hip fracture causes functional decline and increased need for LTC [40, 43]. Osnes et al. [36] and Hektoen et al. [16] are two studies that respectively show the consequences of hip fractures on LTC costs and needs in Norway. Osnes et al. [36] estimate the likelihood for older people living independently in the community of needing LTC after experiencing a fracture to be above 50%. Hektoen et al. [16] calculates the health and care costs in the first year following a hip fracture and find that the greater part of the costs is LTC costs borne by the municipalities. Both studies recommend municipalities to establish hip fracture preventions programs.

### Intervention

In 2007, 15 (of 428) Norwegian municipalities and one district in Bergen implemented the *Safe Aging* program (in Norwegian: Trygge eldre).^3^ The program was initiated by the non-profit organization *Forum for Injury Prevention* (in Norwegian: Skadeforebyggende forum), and the aim of the program was to prevent falls and fall-related injuries among older adults (≥65 years). The program focused especially on reducing hip fractures because of the large associated treatment and quality-of-life costs [40]. Following Lund and Aarö [27], the program used a wide range of measures aimed at changing attitudes, behavior, and the physical and organizational environment. The program was implemented in a non-centralized way and the municipalities could choose which measures to implement and how to design them, the municipalities mainly financed the implemented measures themselves. The project leader of the *Safe Aging* program worked as a coordinator, and supervised contact persons in all participating municipalities. An important part of the program was to support cooperation between municipalities and voluntary activities. In the end, the municipalities focused on similar measures, e.g. information to employees, older adults and relatives, education for employees, physical exercise, preventive home safety assessment and modification help services, and fall registration [40]. We present an overview of the implemented measures in Table 1.

**Table 1:** Intervention description

	Information for older adults	Information for employees	Exercise	Home visits	Fall registration
Namsos	x	x	x	x	x
Fosnes	x		x	x	x
Overhalla	x		x	x	x
Flatanger	x				x
Namdalseid	x	x			x
Hamar	x	x	x		x
Ski	x	x			
Årdal	x		x	x	x
Balestrand	x			x	
Höyanger	x	x		x*	
Förde	x			x*	
Luster	x		x	x*	
Stryn	x		x		
Gloppen	x		x		
Laksevåg	x		x	x	
Lærdal	x				

Notes: * Home visits made by volunteers, not by professionals

All participating municipalities implemented some kind of information measures. The information measures included brochures on safety measures, and the benefits of physical exercise to reduce the risk of hip fractures, either distributed to all households in the municipality or via organizations for retirees and other voluntary organizations. Lectures or courses on the same themes were also held in most of the participating municipalities. Five of the participating municipalities also educated their employees on how to reduce fall-related injuries. Nine municipalities implemented some kind of physical exercise for the at risk population. For the reasonably fit older adults, a wide range of activities were on offer: from weight training at gyms, to Thai-Chi, and exercise in swimming pools. For those in need of more help, living at home or in an institution, exercise groups were held in the institutions or in local health centers. Finally, several municipalities offered preventive home safety assessment and modification help services, where focus was on measures to prevent accidents in the home, but also information about the benefits of physical exercise and information about the supply of preventive measures given in the municipality. Seven municipalities implemented systematic registration of falls in both home services and in institutions. Most of the implemented measures have not stopped at the end of the project period, but are ongoing.

### Data and measures

To measure hip fracture incidence in Norwegian municipalities we collected hospital admissions data from the Norwegian patient registry (NPR) in the period 1999 to 2014. NPR includes, among other variables, information on diagnosis and procedure codes, age, gender, and patients’ municipality of residence for all in and outpatient stays from 1999 [4].

The applied method requires a complete time series in the dependent variables; we therefore dropped municipalities that were involved in mergers in the study period. In 1999, there were 435 municipalities, and of those 14 municipalities were involved in mergers. In addition, we did not have information on hip fracture rates in the districts of Bergen, and since only one city district in Bergen participated in the program, we excluded Bergen from the analysis. Our sample therefore consisted of 420 municipalities, of which 15 were in the treatment group.

There are several ways to quantify hip fracture incidence using hospital registry data. See for example Omsland et al. [35], who compared different methods using registry data. We followed Øien et al., [46], and used the method recommended in Høiberg et al. [18]. Høiberg et al. [18] drew a random sample of admissions from NPR registered with diagnosis and/or procedure codes related to hip fracture, and analyzed the information contained in hospital records for this sample. They found that defining hip fractures according to a combination of diagnosis and procedure codes gives the best correspondence with actual hip fractures as indicated by hospital records. Thus, we defined hip fractures as all hospital admissions coded with a diagnosis code for femoral fracture (ICD10, S72.0–2) and either procedure code (NOMESCO version 1.14) for treatment of femoral fracture (NFJxy, x = 0–9,y = 0–2) or for replacement of hip joint (NFBxy, x = 0–4, y = 0–2; NFB62).

Using this method we calculated the number of hip fractures per 100,000 inhabitants 65 years and older in Norwegian municipalities in the period 1999–2014. We primarily examined this age group since this is the target population for the *Safe Aging* program. However, since the risk of hip fracture is known to be significantly dependent on age and gender, we also calculated separate incidence rates for men and women, and for the age group 80 years and older, to examine potential heterogeneity in the intervention effects based on these factors.

### Study design

We used a difference-in-difference (DD) design to evaluate the effects of the interventions on hip fracture rates. It is well known that the causal assumptions behind the DD method rely on a strong common trends assumption, in that for the estimates to reflect the causal impact of the intervention, both the treated units and the controls would have needed to follow common trends in absence of the intervention (Angrist & Pischke 2011). Using a nearest-neighbor matching algorithm developed by Bonander [5], we therefore identified our control municipalities by matching on pre-intervention trends, which should decrease bias under the assumption that there are no other changes in the post-period that affect hip fracture rates in the analyzed units. After matching, we quantified the average treatment effect on the full treatment group (*n* = 15) in several ways: (1) average post-intervention effects on hip fracture incidence rates per 100.000 person-years, (2) time-varying (dynamic) effects on incidence rates, (3) relative effects (incidence rate ratios) and (4) in the cumulative number of hip fractures prevented during the observed post-period. *P*-values and confidence intervals were calculated assuming a Poisson distribution on the underlying counts. Cluster-robust standard errors were computed by estimating a design effect based on the intra-cluster correlation coefficient in the matched samples, which works well for controlling the false rejection rate in small sample fixed-effects analyses [32]. We also performed a set of subgroup analyses to test for the moderating effects of age group (above 80 years) and sex, and estimated municipality-specific treatment effects due to the variations in interventions implemented by the different municipalities (Table 1). We summarized the relative effect estimates from the latter in an inverse variance fixed effects meta-analysis to formally test for the presence of heterogeneity in the effects using a Q-test [17]. The analysis was performed in R using the idd and metafor packages [5, 44].

## Results

### Descriptive statistics

In Table 2, we present the mean and standard deviation of the number of hip fractures per 100,000 inhabitants across age and gender for treatment municipalities and potential control municipalities in the pre-reform year 2006. As expected, we can see that age and gender are important risk factors for hip fracture. The number of hip fractures in the age group 80 years and older was roughly twofold compared to the number in the whole population 65 years and older. Women were roughly two-thirds more likely to experience a hip-fracture than men in our population.

**Table 2:** Mean and standard deviation of hip fractures per 100.000 across age and sex-specific groups for treatment and potential control municipalities in 2006

Sample			
Age group	Sex	Treatment municipalities (n = 15)	Potential control municipalities (n = 405)
65+ years	Both	1204.21 (320.49)	1196.7 (547.66)
80+ years	Both	2382.33 (870.77)	2688.02 (1454.93)
65+ years	Men	832.68 (405.75)	804.47 (683.9)
80+ years	Men	1683.07 (1366.28)	2070.09 (2714.46)
65+ years	Women	1479.82 (626.27)	1495.61 (832.64)
80+ years	Women	2768.17 (1099.59)	3039.13 (1843.41)

*Notes:* The tables show means and standard deviation of the dependent variables for treatment and potential control municipalities. There are 15 treatment municipalities and 405 potential control municipalities

In 2006, the hip fracture incidence rate at ages 65 and older was fairly similar in treatment municipalities and potential control municipalities. However, the incidence rate at ages 80 years and older, for both genders, was lower in treated municipalities compared to the average of the potential control municipalities. This is somewhat surprising since one would believe that municipalities with high hip fracture incidence rates would to a greater extent implement hip fracture prevention interventions. On the other hand, it could be the case that treatment municipalities, on average, were facing a higher growth in hip fracture incidence rates, and therefore implemented prevention activities to curb this trend. As explained in the previous section, our method deals with differences in levels and pre-intervention trends in the hip fracture incidence rates between treatment municipalities and matched control municipalities, such that these differences should not bias our results.

### Main results

**Fig. 1:**
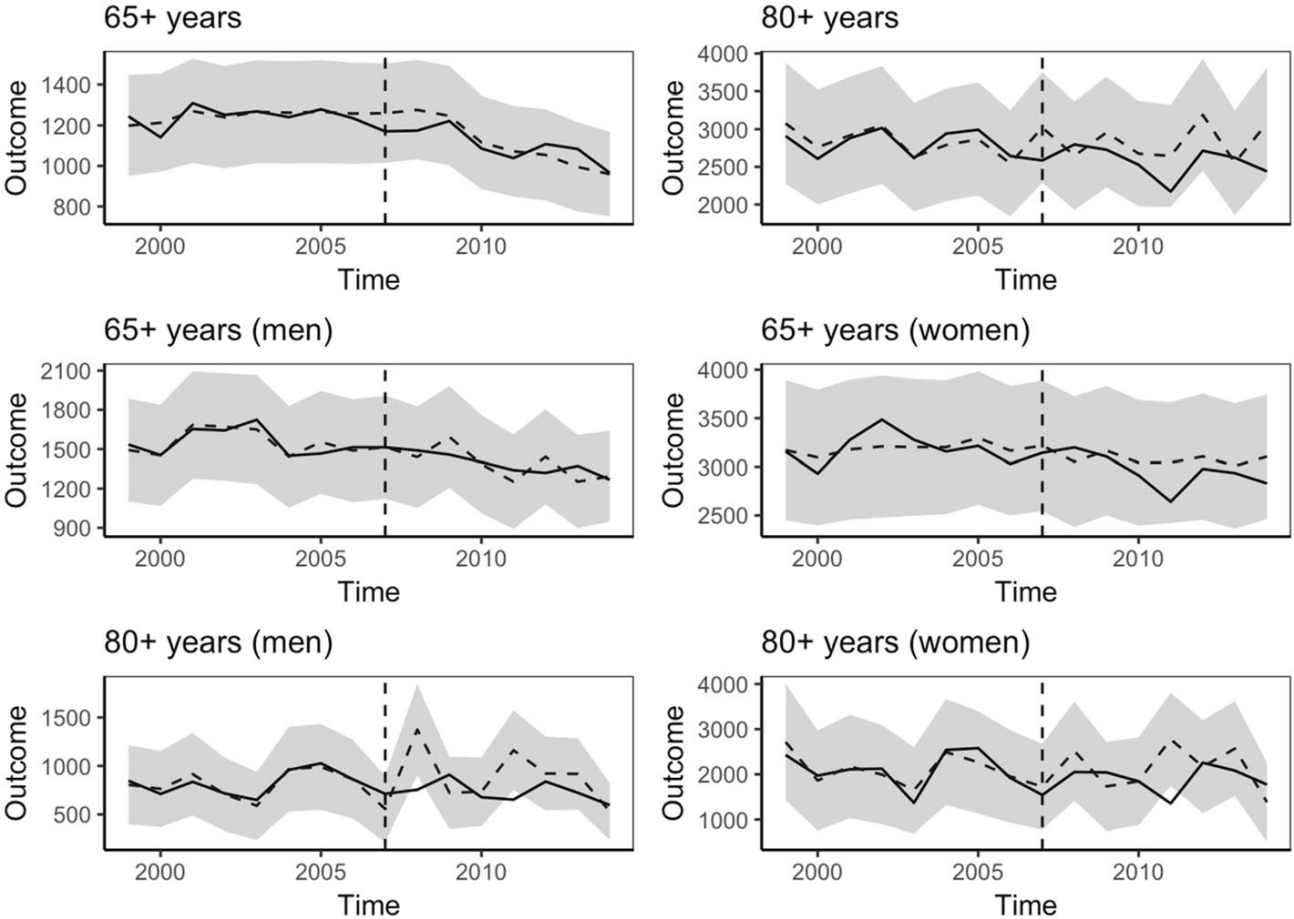
Caption: Trends in hip fracture incidence across age and gender in treatment and matched control municipalities. Notes: Estimated effects of the intervention on the number hip-fractures per 100.000 person-years in Norwegian municipalities in six different subgroups. The solid line is the treatment group, and the dashed line is the control group. The shaded area shows a 95% confidence interval assuming a Poisson distribution

We present the results from the main analysis in the top left panel in Fig. 1. The matched control municipalities followed similar hip fracture trends as the treated municipalities, which speaks for the validity of the effect estimates. As can be seen in Fig. 1, the units did not diverge significantly in the post-period, suggesting that the reductions we see in the treated municipalities also occurred among the controls. Hence, the average intervention effect estimates for the full treatment sample are non-significant and close to zero. The point estimates and confidence intervals are presented in Row 1, Table 3.

**Table 3:** Average effects of the interventions on hip fractures per 100.000 person-years for the entire sample and various age and sex-specific groups

Sample		Effect measure			Controls
Age group	Sex	DD	CE	IRR	n
65+ years	Both	−14.3 (−98.8, 70.3)	−21.6 (− 149.3, 106.1)	0.99 (0.92, 1.06)	4
80+ years	Both	− 274.7 (− 595.2, 45.8)	− 135.8 (− 294.2, 22.7)	0.90 (0.80, 1.02)	1
65+ years	Men	− 131.0 (− 314.0, 52.1)	−90.7 (− 217.4, 36.0)	0.85 (0.66, 1.09)	1
80+ years	Men	− 222.8 (− 705.8, 260.2)	−39.7 (− 125.6, 46.3)	0.85 (0.66, 1.09)	3
65+ years	Women	−1.3 (− 138.3, 140.8)	−1.1 (− 118.1, 115.9)	1.0 (0.91, 1.09)	5
80+ years	Women	− 126.1 (− 422.2, 170)	−39.6 (− 132.5, 53.4)	0.96 (0.87, 1.06)	13

*Notes:* *Significant at the 5%-level. DD = difference-in-differences estimate (in rate per 100.000 person-years), CE = cumulative effect (in number of hip fracture patients), IRR = incidence rate ratio effect. The number of controls to include in each analysis is determined via cross-validation (see text for details)

### Subgroup analysis

As in the main analysis, the matched controls followed similar hip fracture trends as in the treated units across the entire pre-period (Fig. 1; Table 2). Overall, the results from the subgroup analysis were similar to the main analysis in that we found no statistically significant effects for the age group 80 years and above, or in the sex-stratified analyses.

### Municipality-specific treatment effects

**Fig. 2:**
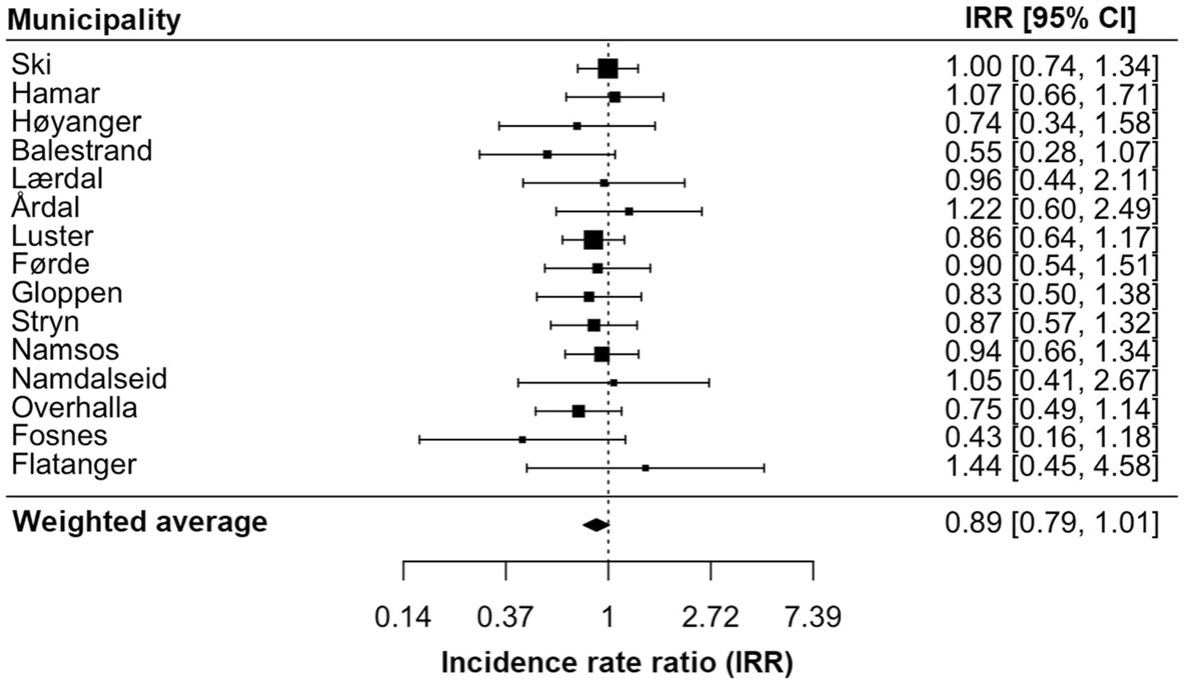
Caption: Forest plot of the estimated municipality-specific relative effects in the 15 treated municipalities. Notes: Point estimates are shown in boxes, along with 95% confidence intervals

We present the results from a meta-analysis of municipality-specific models in Fig. 2. Overall, the effects were clustered around the null, with a non-significant (weighted) average effect of − 11%. There was no significant evidence of heterogeneity, according to a Q-test (Q(df = 14) = 7.8, *p*-val = 0.9).

## Discussion

Overall, we found no convincing evidence of an average effect of the Norwegian *Safe Aging* programs on the incidence of hip fractures in the older population. This result is consistent with previous evaluations of community-based hip fracture prevention programs (Bonander et al., 2008, [29]), so it does not come as a great surprise here. However, we did expect to find some evidence of variation in the municipality-specific effect estimates given that (at least) some of them implemented evidence-based interventions.

As noted in the introduction, this evidence mainly comes from trials at the individual level, and it is therefore unclear how well they translate into practice. An intervention that is rolled out in practice will often deviate substantially from the treatments studied in randomized controlled trials, and interventions must be realized in different local situations [24]. With their many responsibilities, it may be hard for municipal employees to deliver exactly what is expected of them, and the many layers from program initiators down to actual treatment delivery includes many possibilities for the practice to deviate from the program theory. Contextual conditions can moderate the effectiveness of community-based injury prevention programs, causing many to function at a sub-optimal level [34].

In this case, these errors in implementation are apparent in several factors related to the treatments delivered by the treatment municipalities. First, most of the implemented parts of the intervention, e.g. information and education, are not effective in reducing falls according to meta-analyses of randomized trials [11]. It is therefore unlikely that these measures should be effective in the present case. Second, even though home safety assessment and modification may be effective in some settings [11], it seems to be the case that it is important to target risk groups, and that the visits are delivered by an occupational therapist. In the present case, focus has not been on risk groups but the older population in general, and the home safety visits have not solely been conducted by occupational therapists, in some cases volunteers have even conducted them. Additionally, what is included in the Norwegian home safety assessment and modification seem to differ from what have been included in the previously effective ones (Cumming et al. 1999, Nikolaus & Bach 2003, Lin et al. 2007). Overall, it appears that while the efforts by the municipal employees are good-natured and may affect treated individuals, they are not necessarily what is necessary to achieve population-level change in hip fractures. That being said, some of the sub-components of the interventions may have other effects on well-being and health that we could not measure in our data.

### Limitations

There are some limitations to our study that should be mentioned. First, the non-random allocation of intervention municipalities poses a threat to the internal validity of the results. However, our quasi-experimental design specifically matches on pre-intervention trends to find controls that (at least in the pre-period) follow the same trends on the outcome as the treatment units, which decreases the risk of bias. Still, while we are unaware of any other large-scale programs or interventions implemented at the same time, other post-intervention changes that are unrelated to the intervention would bias the results if they affect hip fracture rates in the treatment or control units. Another limitation is that we were unable to measure the actual fidelity of the implementation to that presented in the final report [40], and therefore cannot, without speculation, identify whether the lack of evidence of an effect is due to theoretical failures in the design of the intervention or failures in the implementation of the planned activities. We were not present during the implementation of the interventions, and could therefore not conduct any formal process evaluation during the implementation phase. To our knowledge, no critical assessment of the program theory or its implementation has been conducted. While process evaluation can be done retrospectively [9], the data collection required for a comprehensive analysis of the implementation process was beyond the scope of our current study. We strongly recommend that process evaluation is conducted alongside similar interventions in the future One last limitation we would like to mention is that in aggregated data analysis it is often the case that effect sizes are imprecisely estimated. This is because effects were evaluated for the whole target population, and not only for the population at risk where the treatment is actually expected to have an impact. Therefore, in community trials there is often not enough precision to detect small effects. This is another potential explanation for why the evidence of community interventions is weak.

## Conclusion

There was no evidence of a substantial effect of the implemented interventions on the incidence of hip fractures in any of the studied municipalities. The absence of an effect may be due to low efficacy of the services provided, or of low adherence.

## Footnotes

1 They used the criteria in the “Data Collection Checklist” by the Cochrane Effective Practice and Organization of Care Review Group (EPOC) that are relevant for community interventions.

2 This is regulated in the Health and Care Services Act of 2011.

3 The municipalities are Ski, Hamar, Namsos, Namdalseid, Fosnes, Overhalla, Flatanger, Stryn, Gloppen, Luster, Årdal, Laerdal, Balestrand, Höyanger, Förde, and the city district Laksevåg in Bergen. About 3.3% of the Norwegian population lived in these municipalities in 2007.
